# Exploring the vacuum ultraviolet photochemistry of astrochemically important triatomic molecules

**DOI:** 10.1093/nsr/nwad158

**Published:** 2023-05-27

**Authors:** Yao Chang, Michael N R Ashfold, Kaijun Yuan, Xueming Yang

**Affiliations:** State Key Laboratory of Molecular Reaction Dynamics and Dalian Coherent Light Source, Dalian Institute of Chemical Physics, Chinese Academy of Sciences, Dalian 116023, China; School of Chemistry, University of Bristol, Bristol BS8 1TS, UK; State Key Laboratory of Molecular Reaction Dynamics and Dalian Coherent Light Source, Dalian Institute of Chemical Physics, Chinese Academy of Sciences, Dalian 116023, China; University of Chinese Academy of Sciences, Beijing 100049, China; Hefei National Laboratory, Hefei 230088, China; State Key Laboratory of Molecular Reaction Dynamics and Dalian Coherent Light Source, Dalian Institute of Chemical Physics, Chinese Academy of Sciences, Dalian 116023, China; Hefei National Laboratory, Hefei 230088, China; Department of Chemistry, College of Science, Southern University of Science and Technology, Shenzhen 518055, China

**Keywords:** vacuum ultraviolet photochemistry, photodissociation, free electron laser, interstellar chemistry, chemistry of planetary atmospheres

## Abstract

The recently constructed vacuum ultraviolet (VUV) free electron laser (FEL) at the Dalian Coherent Light Source (DCLS) is yielding a wealth of new and exquisitely detailed information about the photofragmentation dynamics of many small gas-phase molecules. This Review focuses particular attention on five triatomic molecules—H_2_O, H_2_S, CO_2_, OCS and CS_2_. Each shows excitation wavelength-dependent dissociation dynamics, yielding photofragments that populate a range of electronic and (in the case of diatomic fragments) vibrational and rotational quantum states, which can be characterized by different translational spectroscopy methods. The photodissociation of an isolated molecule from a well-defined initial quantum state provides a lens through which one can investigate how and why chemical reactions occur, and provides numerous opportunities for fruitful, synergistic collaborations with high-level *ab initio* quantum chemists. The chosen molecules, their photofragments and the subsequent chemical reaction networks to which they can contribute are all crucial in planetary atmospheres and in interstellar and circumstellar environments. The aims of this Review are 3-fold: to highlight new photochemical insights enabled by the VUV-FEL at the DCLS, notably the recently recognized central atom elimination process that is shown to contribute in all of these triatomic molecules; to highlight some of the potential implications of this rich photochemistry to our understanding of interstellar chemistry and molecular evolution within the universe; and to highlight other and future research directions in areas related to chemical reaction dynamics and astrochemistry that will be enabled by increased access to VUV-FEL sources.

## Teaser

This review summarizes rich photochemistry of triatomic molecules in the vacuum ultraviolet region, and their potential implications to our understanding of interstellar chemistry and molecular evolution in the universe.

## INTRODUCTION

Photodissociation is a branch of photochemistry in which photon absorption results in fission of one or more bonds in a molecule. Photodissociation of oxygen (O_2_) by vacuum ultraviolet (VUV) radiation from the Sun and of ozone (O_3_) at lower altitudes is key to explaining the structure of the ozone layer in Earth's stratosphere. Successful life on Earth relies upon the ozone layer preventing any solar radiation with wavelength λ shorter than 300 nm from reaching Earth's surface.

Most of space has no such protective blanket and molecular photodissociation by radiation with much shorter (e.g. VUV) wavelengths from the Sun has long been recognized as an important source of atoms and small molecular species in, for example, cometary comae. Absorption of a single VUV photon promotes a molecule to a highly excited state from which it may break up promptly (direct dissociation) or more slowly (by predissociation). The resulting photofragments are often much more chemically reactive than the parent precursor, paving the way to (collision-induced) formation of larger molecules. Photodissociation processes are seen as key to understanding and modeling the chemistry prevailing in nearly every type of astrophysical region. Examples include the edges of dense clouds near bright young stars, the surface layers of protoplanetary disks, envelopes around evolved stars and giant molecular clouds. Indeed, such clouds of gas and dust in which photodissociation is the dominant means of molecular destruction are now termed photodissociation or photon-dominated regions (PDRs) [1]. It is in these regions that knowledge of photofragments and their yields is of particular importance. But, as shown in this Review, only now are the broadly tunable sources of VUV radiation with sufficient intensity to undertake the kinds of experiment required to provide such information becoming available.

The burgeoning field of astrochemistry is one, but by no means the only, driver for exploring the VUV photochemistry of small molecules in the gas phase. The photodissociation of an isolated molecule from a well-defined quantum state provides an exquisite lens through which to investigate how and why chemical reactions occur. Comparisons between experimental data and high-level *ab initio* quantum chemical calculations have revealed many fascinating phenomena [2], including quantum interference effects [3,4], striking isotope effects [5,6], and roaming dynamics [7–10], etc.

The last few decades have witnessed numerous photodissociation studies of small molecules following excitation at UV wavelengths [2,7,11]. Studies at VUV wavelengths shorter than that provided by an argon fluoride (ArF) laser (i.e. λ < 193 nm), in contrast, are far fewer in number and largely concentrated at just a few specific wavelengths, e.g. 121.6, 157.6 and 193 nm, due to the lack of a suitably intense, tunable VUV light source. This is a problem, since the electronic absorption of many of the most important small molecules in the early universe, e.g. H_2_, CO, CH_4_, H_2_O, CO_2_, etc., for which detailed photochemical knowledge is essential for modeling the interstellar chemistry lies entirely in the VUV region. Solutions are starting to appear. Tunable VUV sources from table-top lasers, via sum and/or difference four-wave-mixing (FWM) schemes in Kr/Xe gas, have been used to explore aspects of the VUV photodissociation of molecules such as H_2_, CO, N_2_, CO_2_, O_2_, N_2_O, OCS, etc. Such studies have been summarized in recent review papers by Gao *et al.* [12,13]. A free electron laser (FEL), such as that constructed at the Dalian Coherent Light Source (DCLS) [14], offers a very attractive alternative. As shown here, its high brightness and wavelength tunability make it a new and unique tool for state-of-the-art molecular photodissociation dynamics studies across a very broad range of VUV wavelengths.

This review highlights recent VUV photodissociation investigations of selected triatomic molecules (H_2_O, H_2_S, CO_2_, OCS and CS_2_) enabled by the VUV-FEL, emphasizing both the new photodissociation dynamics revealed and the implications of the findings for understanding interstellar and circumstellar environments. These examples provide ample demonstrations of the scientific opportunities offered by use of the FEL: ready access to short photolysis wavelengths (λ < 121.6 nm) and the ease of undertaking studies in an even-handed way over wide wavelength ranges that, in turn, can reveal hitherto unknown fragmentation pathways. Each subsection ends with a short summary of the recent progress and some key remaining questions. The review concludes by outlining several possible exciting future directions and applications of the VUV-FEL.

## EXPERIMENTAL METHODS

The experiments highlighted in this review employed a recently constructed apparatus for molecular photochemistry centered on the VUV-FEL beam line at the DCLS [15,16]. The VUV-FEL output was used to excite molecules directly, accessing dissociative continua and/or predissociating (ro)vibronic levels according to the system and the precise wavelength under study. The resulting photofragments were then detected using a second source of VUV laser radiation, generated using table-top lasers and an appropriate FWM scheme.

**Figure 1.**
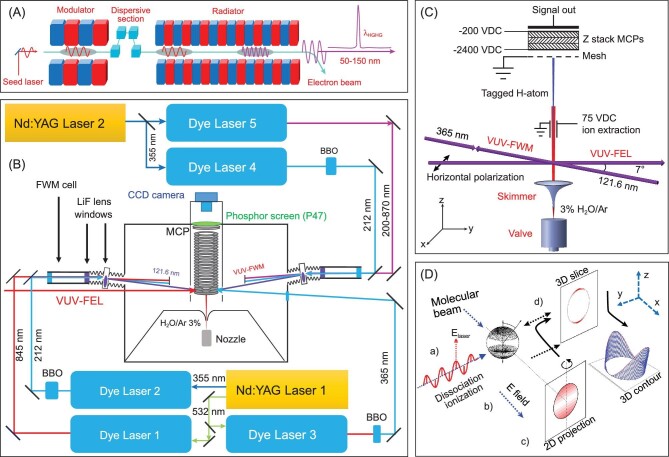
(A) Schematic of the VUV-FEL beamline at DCLS (revised from [15]). (B) The arrangement of the VUV-pump and VUV-probe time-sliced velocity-map imaging (TSVMI) system for photochemistry studies of interstellar small molecules, with the tunable pump radiation derived from either the VUV-FEL or by VUV four-wave mixing (FWM). (C) Schematic showing the molecular beam, laser beam and detection (TOF) axes for photodissociation experiments employing Rydberg tagging methods to detect H(D) atom products (revised from [15]). (D) The VMI approach for measuring photofragment speed and angular distributions (from [20]).

The VUV-FEL facility ran in the high gain harmonic generation mode, wherein the seed laser was injected to interact with the electron beam in the modulator (Fig. 1A). The seeding pulse, in the wavelength (λ_seed_) range of 240–360 nm, was generated from a picosecond duration Ti:sapphire laser pulse. The electron beam, generated from a photocathode radio frequency (RF) gun, was accelerated to a beam energy of ∼300 MeV by seven S-band accelerator structures, yielding a bunch charge of 500 pC. After passing the radiator, the micro-bunched beam was tuned to the *n*-th harmonic of the seed wavelength, leading to coherent FEL radiation with a wavelength of λ/*n*. Optimization of the linear accelerator yielded a high-quality electron beam with emittance of ∼1.5 mm·mrad, energy spread of ∼1‰ and pulse duration of ∼1.5 ps. The VUV-FEL operated at 10–20 Hz and the maximum pulse energy was >500 μJ pulse^−1^ (∼3 × 10^14^ photons pulse^−1^). The horizontally polarized VUV-FEL output (with a typical bandwidth of ∼50 cm^−1^) served as the photolysis laser and entered the photodissociation chamber through a vacuum tube. Every FEL pulse was monitored using an online spectrometer, which established the center wavelength (tunable in the range 50−150 nm).

The tunable VUV radiation for probing the photoproducts was generated by sum or difference frequency mixing (Fig. 1B). In the latter, λ_1_ was set at 212.556 nm (a two-photon resonance in atomic Kr) and λ_2_ was tunable between 220 and 870 nm to yield λ_VUV_ in the range 121–180 nm. Background signals from unwanted secondary dissociation of fragments by the λ_1_ pulse was eliminated by using an off-axis biconvex lithium fluoride (LiF) focusing lens to disperse the λ_1_ and λ_2_ beams away from the photodissociation/photoionization region. Resonance enhanced sum frequency mixing was used to generate VUV-probe wavelengths of <120 nm.

The two independently tunable VUV laser radiation sources were combined in a number of pump (i.e. photolysis)–probe experiments, including H-atom Rydberg tagging time-of-flight (HRTOF) [17,18] and the time-sliced velocity-map ion imaging (TSVMI) [19,20] probe methods. As its name implies, the HRTOF method provides an exquisitely sensitive way of detecting H (and D) atoms. The technique relies on the sequential absorption of photons with wavelengths of 121.6 nm (generated by FWM) and at ∼365 nm (Fig. 1C) to promote H atoms from the ground (*n* = 1) state to excited levels with *n* = 2 and then with *n* ∼ 40. The Rydberg-tagged H atoms are then field ionized just before reaching the detector and recorded by a multichannel plate (MCP). The TSVMI methods (Fig. 1D) were used to detect fragments (atoms, radicals and molecules) other than H atoms. The S(^1^D) photofragments from the photolysis of H_2_S, for example, were probed by one photon excitation at λ = 130.092 nm [21]. The resulting S^+^ ions were accelerated through the remaining ion optics and detected by using a dual MCP detector coupled with a phosphor screen at the end of the ion TOF tube. The transient images on the phosphor screen were recorded using a charge-coupled device camera. Both families of experiments return velocity distributions for the probed fragment, which can be worked up using momentum and energy conservation arguments to yield the total translational energy distribution (*P*(*E*_T_)) of the products.

### Photochemistry of H_2_O

**Figure 2.**
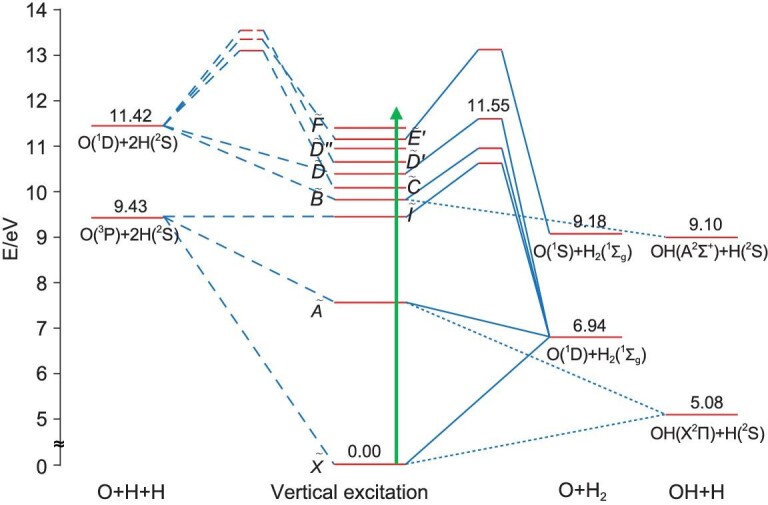
Vertical excitation energies for various excited states of H_2_O featured in this review together with thermochemical threshold energies for the first few O–H bond fission, O atom elimination and three-body dissociation channels, and calculated minimum energy transition states leading to various of these products (from [28]).

Of the >200 detected interstellar molecules [22], water is special. It combines two of the most abundant elements in the universe and it plays a key role in the physics and chemistry of star- and planet-forming regions [23]. On planets, H_2_O and O_2_ are widely acknowledged as essential for potential habitability and the emergence of life. The photodissociation dynamics of H_2_O following excitation to the lower energy singlet excited states (labeled the *Ã*, $\tilde{B}$, $\tilde{C}$ and $\tilde{D}$ states) have been the subject of numerous experimental and theoretical investigations in the past few decades. New quantum phenomena have been revealed and reviewed in [24,25]. Key vertical excitation energies and dissociation limits are summarized in Fig. 2. Here, we focus on recent new and important findings from studies of H_2_O photochemistry at shorter wavelengths (λ < 120 nm, i.e. at energies *E* > 10.2 eV), enabled by the VUV-FEL and VUV-probe pulses generated by FWM.

#### Formation of extremely rotationally excited OH fragments

**Figure 3.**
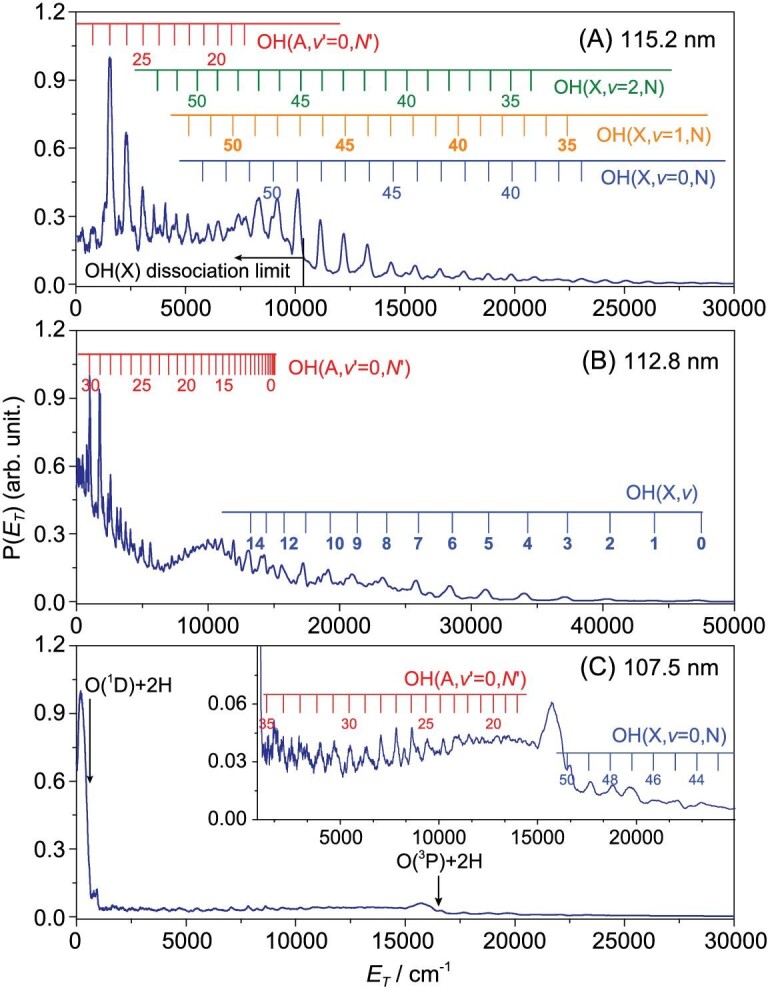
The *P* (*E*_T_) spectra derived from H-atom TOF measurements following photodissociation of H_2_O at (A) 115.2 nm (B) 112.8 nm and (C) 107.5 nm (data from [27,39,44], respectively) with the detection axis in all cases aligned at 54.7^o^ (magic angle) to the polarization direction of the VUV-FEL radiation.

**Figure 4.**
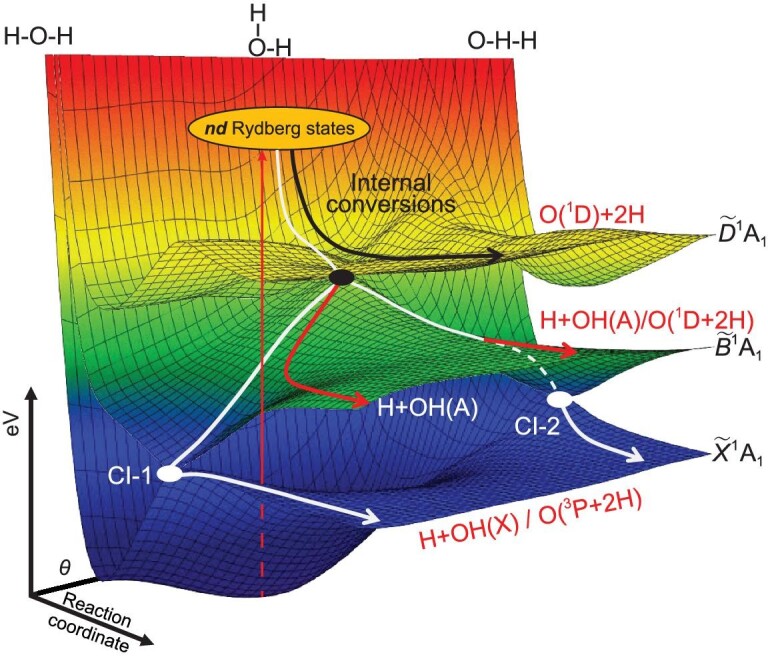
3D representations of potential energy surfaces for the $\tilde{X}$, $\tilde{B}$ and $\tilde{D}$ states of H_2_O plotted as functions of *θ*, the inter-bond angle, and one O–H bond length, illustrating regions of conical intersection (CI) between the $\tilde{D}$ and $\tilde{B}$ states at geometries close to the ground-state equilibrium bond angle and between the $\tilde{B}$ and $\tilde{X}$ state PESs at linear H…OH (CI-1) and OH…H (CI-2) geometries. This representation (adapted from [44]) illustrates vertical excitation to *nd* Rydberg states and subsequent efficient internal conversion to the $\tilde{D}$ state continuum and subsequent bifurcation of the dissociating flux. Some undergo three body dissociation (TBD) to O(^1^D) + 2H products on the $\tilde{D}$ state PES (black arrow) and the remainder undergo non-adiabatic coupling at the CI between the $\tilde{D}$ and $\tilde{B}$ state PESs. Flux that remains on the $\tilde{B}$ state PES can dissociate to electronically excited H + OH(A) and/or O(^1^D) + 2H products (red trajectory) or further couple via CI-1 or CI-2 to access the $\tilde{X}$ state PES and H + OH(X) and/or O(^3^P) + 2H products (white arrows). The potential energy is represented by a false color scale where blue through red represents 0–12 eV.

Ground (X) state OH fragments in rotational levels with quantum number *N* > 49 (and energies in excess of *D*_0_(O–H), the bond dissociation energy of the OH radical) were first observed amongst the products from photodissociation of partially deuterated water (HOD) at 121.6 nm [26]. Similarly rotationally excited OH(X) products were more recently identified following excitation to the $\tilde{D}$ state of H_2_O at λ = 115.2 nm [27]. Figure 3A displays the *P*(*E*_T_) distribution derived from H-atom TOF measurements at this wavelength. The peaks in the spectrum report on the quantum states of the OH partner formed in the dissociation process and the peak intensities reflect the relative populations of these states. As Fig. 3A shows, >30% of the OH(X) photoproducts at this wavelength are formed in rotational levels with energies above *D*_0_(O–H), often in tandem with some vibrational excitation. These OH(X) ‘super-rotors’ only exist by virtue of the centrifugal barrier in the potential energy function associated with the high rotational angular momentum [27]. Only the ground ($\tilde{X}$) and first excited (*Ã*) states of H_2_O correlate adiabatically with H + OH(X) products. Theoretical analysis, guided by the *ab initio* potential energy surfaces (PESs) shown in Fig. 4, shows that H_2_O molecules excited at 115.2 nm dissociate via non-adiabatic coupling at regions of degeneracy (conical intersections (CIs)) between, first, the $\tilde{D}$ and $\tilde{B}$ state PESs, and then between the $\tilde{B}$ and $\tilde{X}$ state PESs [28,29].

The latter degeneracies are key to understanding the massive rotational excitation of the OH(X) products. The topography of the $\tilde{B}$ state PES encourages opening of the ∠HOH bond angle in tandem with elongation of one O–H bond. In the case of H_2_O molecules excited at short VUV wavelengths, momentum conservation and the balance between the bending and stretching forces after non-adiabatic coupling to the $\tilde{B}$ state PES enable sampling of a CI with the $\tilde{X}$ state PES at linear H–O…H geometries. Some molecules undergo further intramolecular ‘orbiting-like’ motion on the $\tilde{B}$ state PES and access a rival CI between the $\tilde{B}$ and $\tilde{X}$ state PESs at linear H…H–O geometries. Both CIs (labeled CI-1 and CI-2, respectively, in Fig. 4) act as ‘funnels’ that facilitate transfer of population to the $\tilde{X}$ state PES and formation of H + OH(X) products, including the observed OH(X) ‘super-rotors’.

The OH(X) radical is commonly observed in the interstellar medium (ISM). Astronomical observations have reported OH(X) radicals carrying extraordinarily high levels of rotational excitation (*N* ≤ 34) in emission from HH 211 [30] and the T Tauri star DG Tau [31]. To the best of our knowledge, there are no mechanisms for forming OH(X) radicals with such high rotational excitation other than VUV photodissociation of H_2_O. This implies that the observation of spontaneous emission from highly rotationally excited OH(X) radicals can act as a proxy for the presence of water in different regions of the ISM. The experimental results described here imply that even more highly rotationally excited OH(X) radicals may well be present in the ISM; their non-observation to date could simply reflect limitations of the available detector [32]. Very recently, these experimental findings have been incorporated to estimate the local UV radiation field and applied to modeling mid-infrared (IR) emission observed from the tip of the HH 211 protostellar jet [33].

Electronically excited OH(A) fragments are also formed in the photodissociation of H_2_O once the photon energy is above its thermochemical threshold (i.e. λ < 136.89 nm, *E* > 9.10 eV, Fig. 2), with a highly inverted rotational state population distribution. The $\tilde{B}$ state of H_2_O correlates adiabatically with H + OH(A) products and the rotational excitation of the latter products can be similarly understood as a carry-over of the opening of ∠HOH in tandem with extension of one O–H bond in molecules that dissociate on the $\tilde{B}$ state PES shown in [34,35]. In one spectacular recent example, H_2_O photolysis at λ = 96.4 nm (a wavelength that populates a Rydberg state lying above the first ionization potential of H_2_O) was shown to yield exceptionally rotationally excited OH(A) fragments, including some in the vibrational ground-state (*v* = 0) level with *N* ≥ 36 [36]. The internal energy of these photofragments is above the thermochemical threshold for the OH(A) → O(^1^D) + H dissociation channel. Again, such extremely rotationally excited products are only bound by their associated centrifugal barrier. These ‘electronically excited OH super-rotors’ can dissociate to O(^1^D) + H by tunneling through the centrifugal barrier, but the tunneling rate is relatively slow. The OH(A) potential is also crossed by no fewer than three repulsive states, each of which correlates with O(^3^P) + H products. Predissociation by coupling to these repulsive states is the dominant decay route for these OH(A) super-rotors. The estimated lifetimes of OH(A, *ν* = 0, *N* = 36 − 40) super-rotors are in the range of 370−57 ps, implying that such electronically excited super-rotors might contribute to chemical reactions in dense atmospheres.

#### Formation of highly vibrationally excited OH fragments

In contrast with the prevalence of rotationally excited OH photoproducts, the identification of highly vibrationally excited OH products from H_2_O photolysis has—until very recently—been rare. The most notable earlier example arose when exciting a few specific low rotational levels of the zero-point (*v* = 0) vibrational level of the $\tilde{C}$ state, which, by virtue of their rotational symmetry, were unable to predissociate by Coriolis (i.e. rotationally induced) coupling to the $\tilde{B}$ state continuum [37,38]. However, recent FEL-enabled studies of H_2_O photolysis at λ = 112.8 nm (Fig. 3B) revealed formation of highly vibrationally excited OH(X) products with an inverted population distribution, maximizing at *v* = 9 and extending to at least *v* = 15 [39]. These OH(X, high *v*) products have been explained by a sequence of non-adiabatic couplings from the photo-prepared $\tilde{E}$ state via an intermediate state of ^1^A_2_ symmetry *en route* to the *Ã* state PES, i.e. the $\tilde{E}$ → $\tilde{I}$^1^A_2_ → $\tilde{A}$ radiationless transitions, mediated by asymmetric stretching motions in regions of configuration space where the respective PESs are near degenerate—i.e. at geometries involving one short and another extended O–H bond. The OH(X, high *v*) products derive from the O–H bond that survives after the final coupling to the *Ã* state PES [38,39].

This finding could also have potentially major astrochemical significance. The OH Meinel bands are important contributors to the airglow in Earth's mesosphere/lower thermosphere [40] and have also been observed in the upper atmospheres of Mars [41] and Venus [42]. The Meinel band emissions are associated with multi-quantum transitions from high to low vibrational levels of the OH(X) radical. The OH(X, high *v*) species in Earth's atmosphere have traditionally been viewed as products of the H + O_3_ reaction, the exothermicity of which allows formation of OH(X) radicals in vibrational levels with *v* ≤ 9 [43]. However, recent modeling shows that OH(X, high *v*) radicals from H_2_O photolysis might make a perceptible contribution to the OH Meinel band dayglow in Earth's upper atmosphere and should dominate in the Martian atmosphere [39].

#### Three-body dissociation to O(^1^D/^3^P) + 2H fragments

The *P*(*E*_T_) spectra derived from TOF measurements of the H atoms formed by H_2_O photolysis at λ = 107.4 nm (Fig. 3C) are dramatically different from those that are dominated by two-body dissociation processes [44]. In addition to weak sharp structures attributable to the H + OH(X) and H + OH(A) channels, the spectrum is dominated by two broad continua: one with *E*_T_ ≤ 600 cm^−1^ that shows a striking angular anisotropy, assigned to the O(^1^D) + 2H three-body dissociation (TBD) channel; and a much broader, weaker feature that spans the range 600 ≤ *E*_T_ ≤ 16 000 cm^−1^, attributable to the O(^3^P) + 2H TBD channel, which displays a much smaller angular anisotropy. Further experiments at λ < 107.4 nm show that O atoms (in their ground (^3^P) and first excited (^1^D) states), rather than OH radicals, are the major O-containing products from H_2_O photolysis at such short wavelengths [44]. Dynamical modeling shows that both TBD processes mostly involve sequential dissociations, via an H + O–H intermediate step. Excitations to *nd* Rydberg states dominate the H_2_O absorption spectrum at these wavelengths and the current consensus is that a significant fraction of the photoexcited H_2_O molecules decay by fast internal conversion to the $\tilde{D}$ state from whence dissociation to O(^1^D) + 2H products is predicted to be barrierless—and thus sufficiently direct to account for the observed recoil anisotropy. A rival non-adiabatic coupling to the $\tilde{B}$ state PES could further boost the O(^1^D) + 2H product yield (via a highly rotationally excited OH(A) intermediate). More importantly, further non-adiabatic coupling via the previously documented $\tilde{B}/\tilde{X}$ CIs offers a plausible route to the observed O(^3^P) + 2H products (via a highly internally excited OH(X) intermediate).

The abiotic generation of molecular O_2_ in planetary atmospheres remains a very active area of investigation [45]. In addition to CO_2_ (see later), water must be considered as another potential source of O_2_, since H_2_O is the third most abundant molecular species in the universe. Recent studies of the coma of comet 67P/Churyumov–Gerasimenko found a strong correlation between the O_2_ and H_2_O signal intensities and that the O_2_/H_2_O ratio was isotropic and did not change systematically with heliocentric distance [46]. TBD is the dominant decay process following excitation of H_2_O at shorter photolysis wavelengths and the TBD yields have been derived from the *P*(*E*_T_) spectra obtained at 12 wavelengths in the range of 92.0 ≤ λ ≤ 121.6 nm (λ = 92 nm defines the onset of the ionization continuum of atomic hydrogen, which limits the penetration of all shorter wavelengths in the ISM) [44]. Convoluting the solar photon flux prevailing in the prebiotic period, the photoabsorption cross sections and the TBD yields implies that ∼21% of the H_2_O photoexcitation events would result in O atoms, which could produce molecular oxygen via the three-body recombination: O + O + M → O_2_ + M. These studies suggest that H_2_O photochemistry might have been an important prebiotic source of O_2_ in Earth's early atmosphere [44]. Astrochemical modeling [47] incorporating these recent photochemical data also suggests that the TBD of H_2_O may be a significant contributor to the O_2_ production rate in the comae of comets at early (<1 day) times following sublimation. Such environments satisfy the requirements of minimal screening of the necessary short-wavelength solar radiation and, at early times, a sufficient collision frequency for the necessary three-body recombination. At longer times, and thus greater distance from the nucleus and lower collision rates, the model predicts O_2_ concentrations close to those obtained when ignoring the TBD channel.

The recent experiments also reveal that more than a third of the O atoms from short-wavelength-induced TBD of H_2_O are formed in the metastable ^1^D state. The O(^1^D) atoms are highly reactive and capable of reacting with almost all the gases emitted into the atmosphere, thereby driving the evolution of the early atmosphere. For instance, the reaction of O(^1^D) with methane could have been a significant source of formaldehyde and methanol in Earth's primitive atmosphere [48]. Further, it is worth recognizing that the O(^1^D) atoms from TBD of H_2_O may contribute to the often monitored O(^1^D → ^3^P) 630.0/636.4-nm emissions in interstellar environments [49].

#### Formation of vibrationally excited H_2_ fragments

H_2_ elimination following VUV photoexcitation of H_2_O is thermodynamically (and spin-) allowed at wavelengths λ <176 nm. The branching ratio for forming O(^1^D) atoms together with ground (X) state H_2_ products at one wavelength (121.6 nm) was measured long ago [50] but any contribution from the higher energy O(^1^S) + H_2_(X) fragmentation channel was only demonstrated for the first time in recent VUV-FEL pump/VUV-FWM probe experiments employing TSVMI detection [51]. O(^1^S) imaging studies following photolysis of H_2_O in the wavelength range λ ∼100–112 nm returned inverted H_2_(X) vibrational-state population distributions peaking at *v* ∼3 or 4, which could be rationalized in terms of the relevant PESs and non-adiabatic couplings *en route* to O(^1^S) + H_2_(X) products. A branching ratio of 16 ± 8% was estimated for this channel [51].

Vibrationally excited H_2_(X, *v*) molecules can survive for several days in rarefied interstellar environments and have been widely observed in the ISM [52]. The vibrational energy can impart greater reactivity (cf. H_2_(X, *v* = 0) molecules), making H_2_(*v*) a reactive species with many atoms, molecules and ions in interstellar space. The C^+^ + H_2_(*v* = 0) → CH^+^ + H reaction, for example, is endothermic with an energy barrier of ∼0.37 eV, but this barrier can be readily surmounted by H_2_(*v* > 0) molecules [53].

Intriguingly, observations towards the reflection nebula NGC2023 reported significantly enhanced emission intensities from H_2_(X, *v* = 4) levels [54]. This matches the maximum in the vibrational-state population of the H_2_ fragments formed via the O(^1^S) + H_2_(X) channel in the short-wavelength photodissociation of H_2_O [51], hinting that H_2_O photolysis could be a contributory source of H_2_(*v*) molecules in PDRs within the ISM. The O(^1^S) atoms observed in the recent VUV-pump–VUV-probe experiments are also very relevant to the ‘forbidden’ atomic oxygen emission lines at 557.7 nm (green) and at 630.0/636.4 nm (red), associated with, respectively, the O(^1^S → ^1^D) and O(^1^D → ^3^P) transitions. These emissions are ubiquitous in terrestrial atmospheres and in cometary comae, where they are viewed as a proxy for H_2_O [55]. A recalculation of the photodissociation rates of both the O(^1^S) + H_2_(X) and O(^1^D) + H_2_(X) channels from solar photolysis of H_2_O based on the recent DCLS measurements returns green-to-red line intensity ratios and intrinsic line widths for both emissions that are consistent with those observed in comets [56].

#### Isotopic effects

Isotope abundance ratios in comets, planets, the solar wind and the ISM are vital to understanding and reconstructing the history and origin of materials in the solar system. Understanding the formation and evolution of water during star and planet formation is key to our recognizing the conditions for life in other planetary systems. Long-standing cometary measurements have returned a wide range of D/H ratios in the water within Jupiter family objects, which tend to rule out the idea that the reservoir is composed solely of Earth-ocean-like water [57]. Most early photochemical models based on the self-shielding effect attributed isotopic fractionation effects to shifts in absorption line positions upon isotopic substitution and did not allow other possible sources of isotopic fractionation in the photodissociation process [58]. Recent experimental studies of CO photodissociation at wavelengths λ ∼93 nm, however, have demonstrated that the branching into electronically excited C atom products is very sensitive to substituting ^12^C by ^13^C [5]. This isotope effect was shown to be sensitively dependent upon the specific rovibronic quantum state that is being excited and has been proposed as a source of isotope heterogeneity.

**Figure 5.**
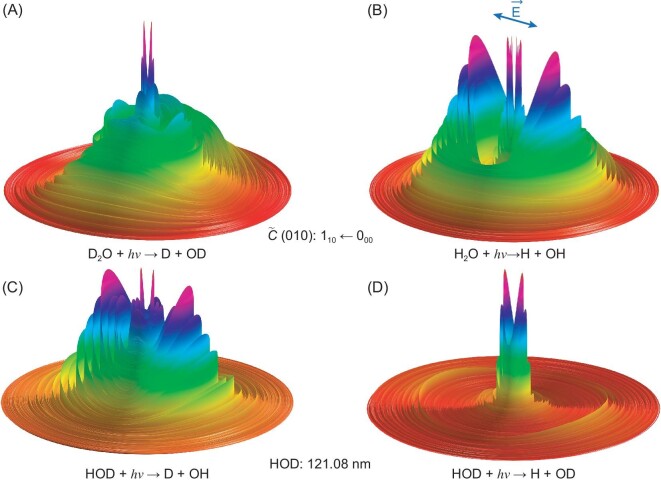
3D contour plots showing the angle-resolved *P* (*E*_T_) distributions of the (A) D + OD products from the photodissociation of D_2_O and (B) H + OH products from the photodissociation of H_2_O via the 1_10_ ← 0_00_ lines of the respective $\tilde{C}$(010)−$\tilde{X}$(000) transitions, with *E*_T_ ≤ 30 000 cm^−1^ (redrawn using data from [6]); and the (C) D + OH and (D) H + OD products from the photodissociation of HOD at 121.08 nm, with *E*_T_ ≤ 20 000 cm^−1^ (redrawn using data from [61]). The double-headed arrow in (B) shows the alignment of the polarization vector of the VUV-FEL radiation, **ϵ**. The outer rings in both plots are associated with the formation of rovibrational states of ground-state OH/OD(X) radical products, whereas the inner structures are mainly due to OH/OD(A) products.

Here we consider how the VUV photochemistry of H_2_O/HOD/D_2_O might influence the D/H ratios of water in comets, planets and the ISM. The 3D contour plots shown in Fig. 5A and B show the *P*(*E*_T_) distributions of the D + OD and H + OH products from photodissociation of, respectively, D_2_O and H_2_O following excitation to specific rotational levels of their respective $\tilde{C}$(010) states at λ ∼122 nm. These studies employed a table-top VUV-FWM photolysis laser, which offers a narrower bandwidth but also much less flexibility than the FEL [6,59]. The action spectra for forming H(D) atoms at these wavelengths and, as Fig. 5A and B shows, the OH(OD) product state distributions all display striking isotopologue-dependent differences. In H_2_O, the probability of coupling to the $\tilde{B}$ state continuum is greatly boosted by an isotopologue-specific accidental vibronic resonance, i.e. $\tilde{C}$(010) → $\tilde{D}$(000) → $\tilde{B}$ → rotationally excited OH(A and X) products [6]. Such accidental-resonance-induced state mixing could contribute to the D/H isotope heterogeneity in the solar system.

Photodissociation processes of the mixed isotopologue, HOD, have also been suggested to play an important role in determining the D/H ratio heterogeneity in the solar system. Photochemical modeling [60] has assumed that HOD photodissociation into the H + OD and D + OH channels shows the same wavelength dependence, but the validity of this assumption has yet to be proven experimentally. Indeed, recent experiments at six different wavelengths in the narrow range of 120.8 ≤ λ ≤ 121.7 nm have returned [H + OD]/[D + OH] branching ratios that vary between 0.70 ± 0.10 at 121.08 nm and 0.49 ± 0.10 at 121.6 nm [61]. Such wide variations raise serious questions about the assumption, particularly at shorter photolysis wavelengths where each isotopologue shows a quite richly structured absorption spectrum. Noting the great abundance of water in the solar nebula, HOD photodissociation must be a potentially significant contributor to D/H isotope heterogeneity and deserves more careful consideration in photochemical models.

As Fig. 5C and D shows, the photodissociation dynamics of HOD at λ = 121.08 nm also show marked channel-dependent differences. For the D + OH channel, most of the OH products are formed in their ground (X) electronic state. Roughly half of these are in the *v* = 0 level, with the remainder distributed over several *v* > 0 levels. These OH(X) products are highly rotationally excited. In the case of the H + OD channel, however, most of the OD products are formed in the *v* = 0 level of the excited (A) electronic state. Of these, >80% are in just three rotational levels, with *N* = 27–29. This localization of the OD(A, *v* = 0) population, termed a ‘single *N* propensity’ when first observed [62], is viewed as a signature of dissociations that explore the near linear OD…H region of the HOD $\tilde{B}$ state PES, whereas the energy disposal in the D + OH(X) products is much more characteristic of molecules that dissociate by non-adiabatic coupling via the $\tilde{B}/\tilde{X}$ CI at linear DO…H geometries [61]. These data provide further illustration of striking isotopic effects in the photodissociation of HOD that could map through as D/H isotopic heterogeneity in the ISM.

#### Summary

The photochemistry of H_2_O has attracted great attention for many years. The new FEL apparatus at DCLS has enabled further investigations, at a hitherto unprecedented level of detail, over a broad range of absorption wavelengths (90 ≤ λ ≤ 120 nm), detecting both H and H_2_ elimination channels. Branching ratios between H and H_2_ fragment formation channels have been reported at specific photolysis wavelengths but determining these branching ratios across the whole VUV region remains a challenge, since it would require quantum yield measurements of all active H_2_O dissociation channels. Accurate determination of wavelength-dependent [H]/[H_2_] branching ratios from H_2_O photolysis is suggested as one of the most important future research directions due to its importance in astrochemical models involving H_2_O.

### Photochemistry of H_2_S

The photodissociation dynamics of H_2_S, the heavier homologue of H_2_O, have also received much attention in recent decades. Solar photodissociation is an important destruction route for interstellar H_2_S molecules [63]. Astrochemical databases currently recommend S–H bond fission, yielding H + SH fragments, as the sole decay process following photoexcitation at all energies up to the first ionization potential [1,64]. Recent VUV-FEL-enabled photodissociation studies of H_2_S have revealed shortcomings in this picture. Since the S atom and SH radical abundances in the ISM are strongly linked with H_2_S, and its photodissociation by VUV photons, a comprehensive understanding of H_2_S photochemistry is clearly desirable for improved astrochemical modeling.

#### The SH(X) radical yield

**Figure 6.**
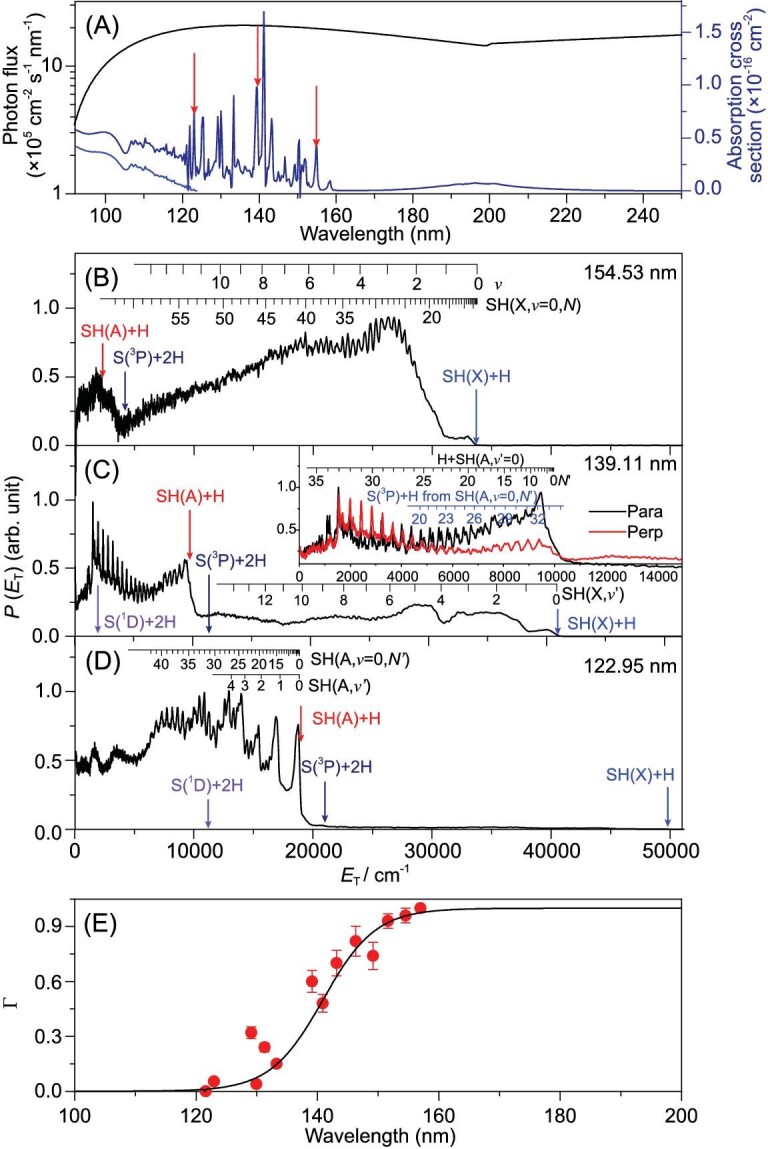
(A) Wavelength dependences of the general interstellar radiation field (ISRF, black line) and the total absorption (σ_tot_, navy line) and photoionization (σ_ion_, blue line) cross sections of H_2_S. The *P*(*E*_T_) spectra derived from H-atom TOF spectra following photodissociation of H_2_S at (B) 154.53 nm, (C) 139.11 nm and (D) 122.95 nm (i.e. when exciting on the features marked with red arrows in (A)), with the detection axis aligned at the magic angle (*θ* = 54.7°) to the polarization vector of the photolysis laser radiation, **ϵ**. The inset in (C) shows an expanded view of the low *E*_T_ part of the corresponding *θ* = 0° and 90° data. The combs on these spectra show the *E*_T_ values associated with formation of H atoms in conjunction with selected rovibrational levels of the primary SH(X) and SH(A) fragments and, in (C), with H atoms formed by predissociation of primary SH(A, *v* = 0, *N*) fragments. The maximum *E*_T_ values associated with the various primary fragmentation channels are shown by colored arrows. (E) The quantum yield, Γ, for forming SH(X) photoproducts (red dots). The sigmoidal function (black line) through these data is used to derive the overall SH(X) product quantum yield ([21]).

As with H_2_O, the electronic absorption spectrum of H_2_S shows continuous absorption at long wavelengths and a structured region, associated with absorption to predissociated Rydberg states, at shorter wavelengths (Fig. 6A). The velocity distributions of the H atom and S(^1^D) atoms formed by FEL-induced photolysis of jet-cooled H_2_S molecules have been recorded at many wavelengths in the range 122 ≤ λ ≤ 155 nm [21]. S(^1^S) photoproducts have also been detected, by TSVMI, following VUV-FEL excitation in the range 122 ≤ λ ≤ 136 nm [65]. The *P*(*E*_T_) spectra derived from HRTOF measurements at three widely separated wavelengths, shown in Fig. 6B–D, display a clear evolution. Long-wavelength excitation yields ground-state SH(X) fragments in a very broad spread of rovibrational levels; the quantum yield for forming H + SH(X) products is unity at λ ≥ 157.6 nm. Electronically excited SH(A) products become increasingly important as the photolysis wavelength is reduced, however, and are the exclusive SH product at λ = 122.95 and 121.6 nm. Formation of SH(A) super-rotors is evident in Fig. 6C and D, and can be explained by non-adiabatic coupling to and dissociation on a PES with a topography reminiscent of that of the $\tilde{B}$ state of H_2_O [21]. Since the TBD channel to S(^1^D) + 2H products is evident in the *P*(*E*_T_) spectra derived from both the H-atom TOF measurements and the S(^1^D) ion images recorded at shorter wavelengths, this component serves as a reference when estimating branching ratios for the H and H_2_ forming product channels. These experimental data returned a quantum yield for forming H_2_ + S(^1^D) products at λ = 139.11 nm of ≤0.12. The quantum yield for S(^1^S) + H_2_ production is estimated to be smaller still [65], implying that S–H bond fission is the dominant primary event at this wavelength [21].

However, all SH(A) primary products will predissociate on a nanosecond (or shorter) timescale to yield another H atom (along with an S(^3^P_J_) partner) [66]. This secondary dissociation contributes another progression in the *P*(*E*_T_) spectrum derived from HRTOF measurements—as illustrated in the case of the λ = 139.11 nm data in the inset within Fig. 6C. Thus, only those H_2_S photodissociation events that yield SH(X) radical products should contribute to the SH/H_2_S abundance ratios observed in different regions of the ISM. Convoluting the wavelength-dependent H_2_S absorption, the spectrum of the general interstellar radiation field (ISRF) (Fig. 6A) and the quantum yield for forming SH(X) radicals determined at each wavelength investigated (Fig. 6E) reveals that only ∼26% of H_2_S molecules excited by the general ISRF would yield a stable SH radical [21]. This experimental result may explain the interstellar SH/H_2_S abundance ratio of ∼13% reported from the star-forming region W49N [67] and is generally consistent with more recent model predictions that suggest an [SH]/[H_2_S] ratio of ≤0.2–0.6 at the edge of PDR regions in the ISM [68]. This quantum yield estimate implies that atomic sulfur is the dominant S-containing species from H_2_S processing by the ISRF—a result that accords well with the observed strong correlation between measured S and H_2_S signals in the coma of comet 67P/Churyumov–Gerasimenko [69].

The S-atom abundances can also provide clues about star-formation history, connecting the local and distant universes [70]. There are several S-atom transitions available for observational studies in the stellar spectra, including the ‘forbidden’ S(^1^D) → S(^3^P_2_) transition at 1082 nm [71,72]. As one of the most abundant S-bearing molecules in many interstellar environments, H_2_S photolysis might be an important source of the observed S(^1^D) atoms—a proposal that might be amenable to testing by careful linewidth measurements.

#### Parent rotational state-dependent predissociation dynamics

This subsection provides a further striking illustration of the richness and complexity of the H_2_S photodissociation process. Figure 6C displays the *P*(*E*_T_) spectrum obtained from the HRTOF measurements following the FEL excitation of a jet-cooled H_2_S sample at λ ∼139.1 nm, which lies in the middle of the VUV wavelength range where H, SH(X), SH(A), S(^1^D) and H_2_ fragments have all been observed [21]. The bandwidth of the FEL pulse ensured that the experiment sampled much of the origin (i.e. *v* = 0 ← *v* = 0) band of the ^1^B_1_ ← $\tilde{X}$^1^A_1_ transition. This ^1^B_1_ Rydberg state predissociates (by non-adiabatic coupling to dissociative valence excited states) at a rate that is sufficiently slow that many of its rotational (*J_KaKc_*) levels contribute resolvable fine structure within the ^1^B_1_ ← $\tilde{X}$^1^A_1_ band.

**Figure 7.**
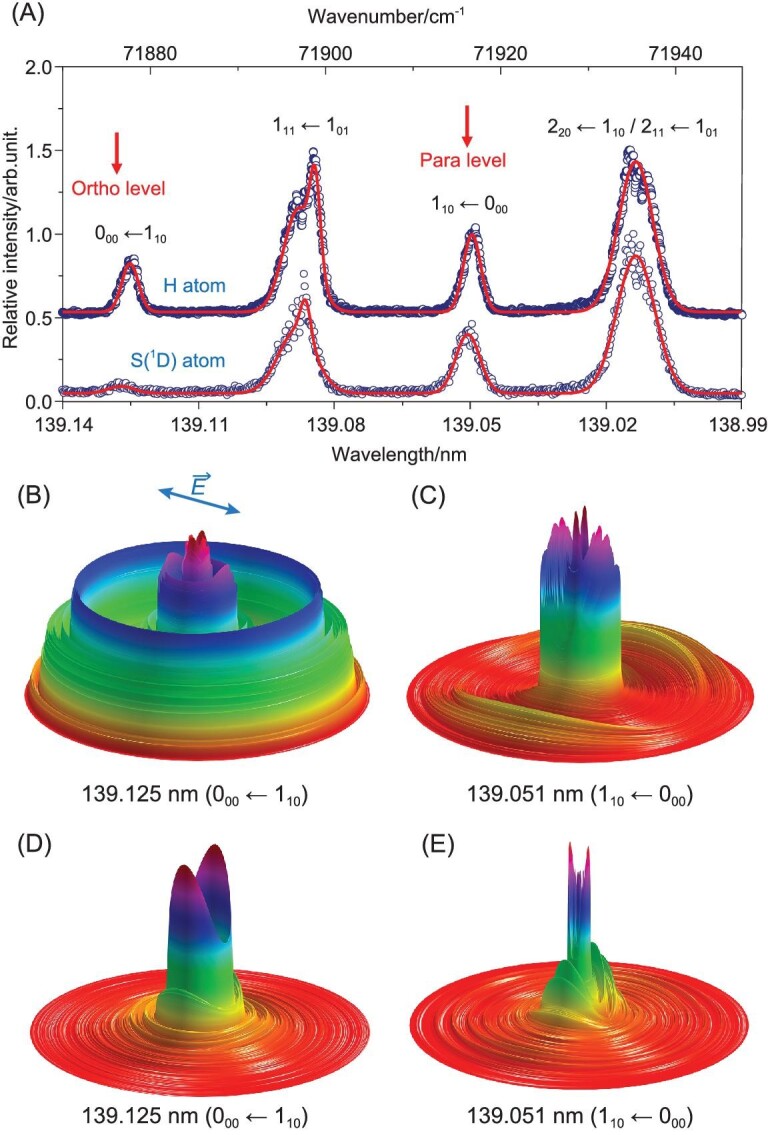
(A) The H-atom (upper curve) and S(^1^D)-atom (lower curve) PHOFEX spectra obtained following photodissociation of a jet-cooled H_2_S in Ar sample at wavelengths in the range 139.14 ≥ λ ≥ 138.99 nm, offset vertical by 0.5-arb. units for clarity. The dominant transitions contributing to the four features are indicated (revised from [19]). The 3D contour plots of the *P*(*E*_T_) distributions of the H + SH products from the photodissociation of H_2_S via (B) the 0_00_ ← 1_10_ and (C) the 1_10_ ← 0_00_ lines, with *E*_T_ ≤ 41 000 cm^−1^. Analogous 3D contour plots of the S(^1^D) + H_2_ products when exciting on (D) the 0_00_ ← 1_10_ and (E) the 1_10_ ← 0_00_ lines at ∼139 nm, with *E*_T_ ≤ 35 000 cm^−1^ (data in all cases from [19]). The double-headed arrow in panel B shows the alignment of **ϵ**. The outer rings in plots (B) and (C) are associated with the population of rovibrational levels of the ground-state SH(X) radical products, whereas the inner structures are mainly due to SH(A) and/or S(^3^P) + 2H products. The rings in plots (D) and (E) are associated with the formation of rovibrational states of the H_2_(X) products.

This is illustrated in Fig. 7A, which shows the photofragment excitation (PHOFEX) spectra for forming H atoms and S(^1^D) atoms recorded by the HRTOF and TSVMI techniques, respectively, using a much narrower bandwidth VUV FWM pump laser source [19]. The spectra show four well-resolved features, associated with transitions to specific rotational levels of the ^1^B_1_(*v* = 0) state, but the relative line intensities are obviously different [19]. Earlier spectroscopy studies [73] of this ^1^B_1_ state had identified both homogeneous (i.e. vibronic) and heterogeneous (i.e. Coriolis- or rotationally induced) predissociation mechanisms but were silent with regard to the products. The rate of Coriolis-driven predissociation was shown to scale with <*J_b_*^2^> (i.e. with the expectation value of the square of the angular momentum about the *b*-inertial axes in the excited rotational level), indicating that this predissociation pathway involved coupling to a continuum of ^1^A′ symmetry.

The 3D contour plots shown in Fig. 7 highlight the very different *P*(*E*_T_) distributions obtained from the HRTOF (panels B and C) and S(^1^D) imaging (panels D and E) data when exciting at, respectively, λ = 139.125 nm (panels B and D) and λ = 139.051 nm (panels C and E). The former involves a single rovibronic transition (the 0_00_ ← 1_10_ line). The populated level has <*J_b_*^2^> = 0 and decays by a pure homogeneous predissociation pathway yielding S(^1^D) + H_2_(X, high *v*, low *J*) and/or 2H fragments (Fig. 7D) and H + SH(X) fragments in a very broad range of *v, N* levels (Fig. 7B) products following vibronic coupling to a dissociative valence continuum of ^1^A″ symmetry. Excitation at λ = 139.051 nm, in contrast, is dominated by the 1_10_ ← 0_00_ transition. The excited level in this case has <*J_b_*^2^> = 1 and the additional heterogeneous decay route by Coriolis coupling to the ^1^A′ continuum can contribute, resulting in S(^1^D) + H_2_(X, low *v*, high *J*) (Fig. 7E) and H + SH(A, low *v*, high *N*) (Fig. 7C) products. As noted above, the SH(A) products predissociate further, to H + S(^3^P) atoms. The two other peaks in the PHOFEX spectrum are blended features involving more than one rovibronic transition. The photoexcited levels populated when exciting either feature can each decay by both predissociation pathways, but with different relative rates, so the measured *P*(*E*_T_) spectra are sensitively dependent on the exact excitation wavelength, the sample temperature and thus the relative populations of the various H_2_S($\tilde{X}$, *v* = 0, *J_KaKc_*) levels [19].

These studies reveal another important and fundamental detail that is not immediately evident in the *P*(*E*_T_) spectra plotted with the compressed scale used in Fig. 7. H_2_S molecules contain two identical H nuclei (fermions) and symmetry dictates that each rotational level of H_2_S must satisfy either *ortho*- or *para*-nuclear spin statistics. In the case of H_2_S, these are distinguished by whether the sum *K_a_* + *K_c_* in the ground state is, respectively, an odd or even number. Key to the current discussion, the 0_00_ ← 1_10_ (λ = 139.125 nm) and 1_10_ ← 0_00_ (λ = 139.051 nm) transitions sample, respectively, *ortho*- and *para*-H_2_S molecules and analysis of the *P*(*E*_T_) spectra of the resulting S(^1^D) + H_2_(X, *v, J*) photoproducts reveals rigorous conservation of nuclear spin symmetry in the dissociation process: *ortho*-H_2_S molecules yield only *ortho*-H_2_ (i.e. odd *J*) products and *para*-H_2_S molecules yield only *para*-H_2_ (i.e. even *J*) products.

#### Summary

These recent studies of H_2_S photolysis provide some of the most complete experimental investigations of molecular photofragmentation processes reported to date, affording initial parent quantum state selection and detailed investigation of competing product channels. After H_2_O, H_2_S is probably currently the second most comprehensively studied molecule. Its photodissociation dynamics have been studied across the whole range of VUV wavelengths up to the first ionization limit and all spin-allowed dissociation channels have been explored. The recent experimental studies afford detailed views of different photofragmentation pathways in H_2_S, but any complete interpretation of the dynamics still requires better knowledge of the topographies of, and non-adiabatic couplings between, the various excited-state PESs.

### Photochemistry of CO_2_, OCS and CS_2_

Carbon dioxide (CO_2_) is an important molecule in Earth's atmosphere, as one of the main greenhouse gases. It is also the major (>95%) component in the atmospheres of Venus and Mars and, as noted earlier in this Review, is generally considered the most likely source of molecular oxygen in the early prebiotic atmosphere of Earth [74]. Carbonyl sulfide (OCS) is the most abundant, long-lived S-containing gas in Earth's atmosphere and a major supplier of sulfur to the stratospheric sulfate aerosol layer. OCS has been observed in the deep atmosphere of Venus, in dense molecular clouds and in Jupiter's atmosphere following the impact of comets [75]. Carbon disulfide (CS_2_) is also ubiquitous in the ISM and has been proposed as a likely precursor for CS and S_2_ radicals observed in cometary coma [76].

Unsurprisingly, therefore, the photochemistry of these molecules has been the subject of many previous theoretical and experimental studies. Each of the molecules has 16 valence electrons in the ground state and a linear ground-state equilibrium geometry. Excitation at longer UV wavelengths in each case induces a π* ← π electron promotion and results in population of one or more bent excited valence states. As in H_2_O and H_2_S, the absorption spectrum of each of these molecules at shorter (VUV) wavelengths is dominated by sharp features associated with excitation to predissociated Rydberg states.

#### Photochemistry of CO_2_

The electronic spectrum of CO_2_ displays two weak and partially overlapping regions of continuous absorption in the wavelength range 120 < λ < 180 nm. The long-recognized primary photodissociation following VUV photoexcitation involves single C–O bond fission. Several studies at λ = 157.6 nm have reported on the competing spin-forbidden and spin-allowed fragmentation paths to, respectively, O(^3^P_J_) and O(^1^D) atoms together with ground-state CO(X) products [77]. More recent TSVMI studies, detecting the O(^3^P_J_) atom products, have provided detailed information about the CO(X, *v*) vibrational-state population distributions and the product recoil anisotropies following excitation at shorter wavelengths in the range 129.02 ≤ λ ≤ 134.67 nm [78].

Almost all of the energy-allowed C–O bond fission channels correlating with O(^3^P_J_), O(^1^D) and O(^1^S) products have now been confirmed following excitation of CO_2_ in the wavelength ranges 90.67 ≤ λ ≤ 91.58 nm [79] and 101.47 ≤ λ ≤ 103.76 nm [80], and the O(^1^D) and O(^1^S) forming channels have been investigated further by VUV-FEL-enabled TSVMI measurements in the range 104.76 ≤ λ ≤ 108.82 nm [16,81]. The CO(X) partner fragments are formed highly vibrationally excited, populating *v* levels up to the limit dictated by the available energy. Such data provide valuable information about the photodissociation mechanisms of CO_2_ that can be used to test and validate future higher-level *ab initio* calculations.

**Figure 8.**
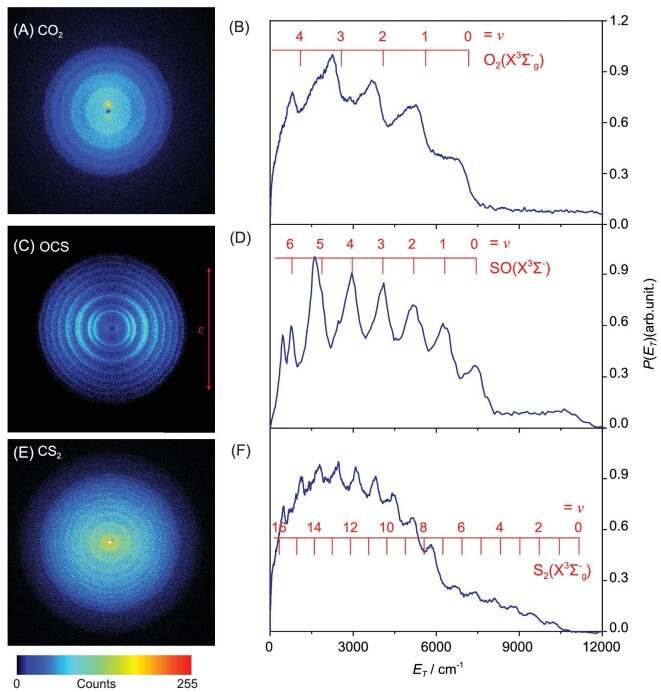
Time-sliced ion images and the corresponding *P* (*E*_T_) spectra from: (A) and (B) the photodissociation of CO_2_ at λ = 100.7 nm obtained by monitoring the C(^3^P_2_) products (VUV-FEL photolysis and VUV + UV 1 + 1’ ionization detection); (C) and (D) the photolysis of OCS at λ = 126.1 nm, detecting the C(^3^P_0_) products (VUV-FWM photolysis and VUV + UV 1 + 1’ ionization detection, data adapted from [87]); and (E) and (F), the photodissociation of CS_2_ at λ = 141.0 nm detecting the C(^3^P_1_) products (VUV-FEL photolysis and VUV + UV 1 + 1’ ionization detection, data adapted from [91]). The rings evident in the images (and the peaks in the corresponding *P* (*E*_T_) spectra) report on the population of different vibronic (*v*) states of the O_2_, SO and S_2_ co-products, as indicated by the superposed combs.

Much less expected was the finding of a direct channel yielding molecular oxygen, i.e. elimination of the central atom, to yield C(^3^P_J_) + O_2_(X) products when exciting in the range 101.5 ≤ λ ≤ 107.2 nm [82]. Figure 8A shows the *P*(*E*_T_) spectrum derived by imaging the C(^3^P_2_) products, following CO_2_ photolysis by the VUV-FEL at λ = 100.7 nm, which reveals population of all energetically available O_2_(X, *v*) partner states. The O_2_ elimination from dissociative electron attachment to CO_2_ has also been reported recently [83]. Both processes could be of great importance for understanding the origin of O_2_ in Earth's prebiotic primitive atmosphere, in which CO_2_ is believed to have been a major component.

#### Photochemistry of OCS

As with CO_2_, the electronic spectrum of OCS shows diffuse absorption bands attributable to excited valence states and sharper Rydberg features at shorter VUV wavelengths. The photodissociation of OCS at near UV wavelengths λ >200 nm is dominated by the spin-allowed channel yielding S(^1^D) + CO(X) products; the latter are formed vibrationally ‘cold’ but rotationally ‘hot’, in at least qualitative accord with that expected given the forces introduced by a bent ← linear excitation process [84]. The same product channel has been investigated using TSVMI methods following excitation in the range 142.98 ≤ λ ≤ 154.37 nm [75] and other single-bond fission channels yielding S(^1^S) + CO(X) [85] and S(^3^P) + CO(A) [86] products have also been reported when exciting at similar VUV wavelengths.

Figure 8B illustrates the operation of a C(^3^P_0_) atom elimination channel yielding SO(X, *v*) co-fragments (analogous to the CO_2_ → C(^3^P) + O_2_(X) process reported above) when exciting OCS at λ = 126.1 nm. This channel was shown to be active at all wavelengths investigated in the range 126.1 ≤ λ ≤ 138.5 nm and, in all cases, to yield SO(X) fragments in all vibrational states permitted by energy conservation [87].

#### Photochemistry of CS_2_

The photodissociation of CS_2_ following excitation to the strongly absorbing excited state of ^1^B_2_ symmetry at λ ∼200 nm has been studied using a range of techniques, which have shown that both the S(^3^P) + CS(X) and S(^1^D) + CS(X) fragmentation channels are active but return some wide differences in branching ratios [88,89]. Electronically excited CS(a^3^Π) photoproducts are observed once with λ < 158 nm and are the dominant diatomic photoproduct in the region 125 ≤ λ ≤ 150 nm [88]. Contemporary studies of the associated dissociation dynamics are limited to a TSVMI study of the S(^1^D) and S(^3^P_J_) products formed following two-photon absorption at four wavelengths at the low-energy end of this range (303 ≤ λ ≤ 315 nm) [90]. As with CO_2_ and OCS, C(^3^P) atom elimination has also been demonstrated from CS_2_ following two-photon excitation in this wavelength region and using one VUV-FEL photon in the range of 127.9 ≤ λ ≤ 143.9 nm [91]. As Fig. 8C shows, the S_2_ partners are deduced to be formed in a broad spread of vibrational levels of the triplet ground (X) state and, when energetically allowed, in the excited a^1^Δ and b^1^Σ^+^ states.

CS and S_2_ are both observed in the coma of comets and studies of 67P/Churyumov–Gerasimenko show that CS is a daughter of CS_2_ [92]. The source of the observed S_2_ species is less clear. The S_2_ species has a short lifetime in the gas phase, as it is readily destroyed by UV excitation at λ ∼283 nm [1]. Thus it is excluded from some astrochemical models and in other instances, as in the case of the coma of 67P/Churyumov–Gerasimenko, its observed yield is much greater than the model predictions [76]. This could imply a hitherto unrecognized production route, i.e. that CS_2_ photolysis might contribute S_2_ daughter species in the ISM.

#### Summary

Notwithstanding impressive recent progress towards identifying active fragmentation channels in the VUV photolysis of the molecules summarized in this section, the wavelength-dependent product branching ratios in almost all cases remain to be characterized. From an astrophysical perspective, these are arguably the most important information, particularly in the case of CO_2_ given its central importance in astrochemistry and planetary science. In this regard, we highlight the estimation of the relative yields of the C(^3^P_J_) + O_2_(X) vs. O(^1^S) + CO(X) product channels (5 ± 2%) following CO_2_ photolysis in the range 101.5 ≤ λ ≤ 107.2 nm, i.e. at energies just above the energetic onset of the former process, from measurements of relative signal intensities and consideration of the relative detection efficiencies [82].

The identification of the central atom elimination channel—wherein two chemical bonds break and a new one is formed—in all three of these molecules suggests that such processes may be much more general than recognized hitherto. Indeed, the earlier sections reported observations of O(^1^D) + H_2_ and S(^1^D) + H_2_ channels from VUV photodissociation of H_2_O and H_2_S, respectively.

The recent literature outlines two different mechanisms for such photoinduced central atom eliminations. One invokes dissociation from a transition state accessed by prior structural rearrangements of the molecule, the other invokes fission of one chemical bond followed by an intramolecular ‘reaction’ between the resulting fragments—a process often termed ‘roaming’ [10,93]. Recent theoretical studies of C(^3^P) elimination from CS_2_, for example, have identified local minima associated with both linear CSS and cyclic CS_2_ structures on the ground-state PES and non-adiabatic pathways—typically involving the second ^1^B_2_ PES accessible at energies above ∼7.2 eV—via which such structures might plausibly be sampled and could aid C(^3^P) elimination [94]. This would be viewed as an example of the transition-state mechanism and it remains unclear to what extent the ‘classic’ roaming mechanism, such as that identified for the UV photodissociation of H_2_CO [93], will contribute in the photodissociation of triatomic molecules. A roaming mechanism has been invoked to account for the almost isotropic recoil velocity distributions observed for the C(^3^P) + SO(X) products formed when exciting OCS just above the energetic threshold for this process, but the photo-elimination dynamics observed at shorter excitation wavelengths are more readily accommodated by a sequence of non-adiabatic couplings between PESs. The orbiting motion of an H atom about the OH (or SH) partners invoked in the photodissociation pathways of H_2_O (H_2_S) that sample the CIs at linear H…H–O (H…H–S) geometries might also be viewed as an example (all be it a very constrained example) of a roaming mechanism. In general, however, the threshold for eliminating the central atom in a triatomic molecule will lie at high energies (i.e. in the VUV region) and the density of non-adiabatic couplings between many PESs will make it hard to distinguish roaming- and transitional-state pathways to the same products.

## OUTLOOK

The foregoing reports just a fraction of the molecules for which the VUV-FEL light source has enabled new and detailed photodissociation dynamics studies, focusing particularly on triatomic systems of importance in planetary atmospheres and in interstellar and circumstellar environments. The chosen examples demonstrate many of the unique attributes of an FEL source for studies of this kind. It affords ready availability of short (λ < 121.6 nm) photolysis wavelengths (tunable down to λ ∼ 50 nm if required), simplifies studies of different target molecules over wide wavelength ranges in an even-handed way and thereby facilitates the identification of hitherto unrecognized fragmentation pathways.

Photodissociation induced by the VUV-FEL can also provide a pulsed atomic beam source for inelastic and reactive gas-phase and gas-surface scattering studies. UV photolysis of HI(DI) molecules within their A-band continuum (200 ≤ λ ≤ 270 nm) has long been a popular route for forming H(D) atom reactants with well-defined, narrow-velocity (and kinetic-energy) distributions [95,96]. Moving to slightly shorter photolysis wavelengths (e.g. 193 nm) has offered a route to forming H(D) atoms with higher translational energies, boosting the prospects for observing new insights into non-adiabatic reaction dynamics involving excited-state PESs (e.g. studies of geometric phase effects in the H + HD → H_2_ + D reaction [97]) and for studies of the inelastic scattering of H atoms off metal and graphene surfaces [98], but the advent of the VUV-FEL at DCLS opens the way to the first well-defined studies of such processes at much higher, hyperthermal, translational energies.

Reactions between neutral gas-phase species have long been recognized as important in molecular evolution in extraterrestrial environments. Reactions involving radical species are now seen as key to driving the growth of more complex organic molecules and solar photolysis is one of the most important ways of generating such radicals. The potential astrochemical significance of VUV-FEL-induced molecular photodissociation studies such as those surveyed in this Review thus extends beyond the revelation of sources of smaller photofragments. Such photolyses can also serve as well-defined sources of atoms and radicals (in their ground and/or excited states) for studies of collision-induced growth of larger molecules via reactions with other species.

To date, the VUV-FEL at DCLS has typically been run with picosecond pulse durations, but it is designed to operate also as an ultrafast light source capable of delivering pulse durations as short as ∼100 fs. Such VUV-FELs can be used as a probe laser to capture the ultrafast dynamical process occurring during a molecular dissociation or isomerization. One recent illustration used extreme UV (XUV) radiation at λ = 64.44 nm (from the FEL at FERMI) and time-resolved photoelectron spectroscopy (TRPES) methods to track the ultrafast photochemistry of a heterocyclic molecule, thiophenone, during and after UV-induced ring opening [99]. The wavelength range and the ease of tuning the VUV-FEL at DCLS offers a ready route to ionizing essentially all molecules, opening the way to preparing state-selected molecular cations (e.g. by appropriate threshold ionization), to distinguishing isomers or different sizes of cluster (by differences in their respective ionization thresholds) [100] and to probing ultrafast reaction dynamics (e.g. by TRPES measurements, as in the recent thiophenone ring-opening study).
